# Curcumin suppresses osteogenesis by inducing miR-126a-3p and subsequently suppressing the WNT/LRP6 pathway

**DOI:** 10.18632/aging.102232

**Published:** 2019-09-03

**Authors:** Hongling Li, Lifeng Yue, Haoying Xu, Na Li, Jing Li, Zhiguo Zhang, Robert Chunhua Zhao

**Affiliations:** 1 Institute of Basic Medical Sciences Chinese Academy of Medical Sciences, School of Basic Medicine Peking Union Medical College, Peking Union Medical College Hospital, Beijing Key Laboratory of New Drug Development and Clinical Trial of Stem Cell Therapy, Beijing 100005, China; 2 Beijing Dongzhimen Hospital, Beijing University of Chinese Medicine, Beijing 100700, China; 3 Institute of Basic Theory, China Academy of Chinese Medical Sciences, Beijing 100700, China

**Keywords:** mesenchymal stem cells, curcumin, osteogenesis, miR-126a-3p, LRP6

## Abstract

Curcumin, a natural phenolic biphenyl compound derived from the plant *Curcuma longa,* modulates multiple steps of carcinogenesis partly by affecting the expression of miRNAs. Interestingly, cancer development shares many of the same signalling pathways with bone formation. Reduced bone mass creates favourable conditions for tumor metastasis. However, the effects and mechanism of curcumin on bone formation and osteogenesis are relatively unknown and controversial. We demonstrated that curcumin inhibited osteogenesis of human adipose-derived mesenchymal stem cells (hADSCs) in a concentration-dependent manner. In hADSCs, curcumin modulates the expression of a series of miRNAs, including miR-126a-3p, during osteogenesis. Overexpression or inhibition of miR-126a-3p is required for the effect of curcumin on osteogenesis. Further investigation indicated that miR-126a-3p directly targets and inhibits LRP6 through binding to its 3’-UTR, and then blocks WNT activation. Our findings suggest that the use of curcumin as an anti-tumor agent may lead to decreased bone mass through the suppression of osteogenesis. Knowing whether the long-term or high doses use of curcumin will cause decreased bone mass and bone density, which might increase the potential threat of tumor metastasis, also requires a neutral assessment of the role of curcumin in both regulating bone formation and bone absorption.

## INTRODUCTION

Curcumin is a well-known dietary polyphenol derived from the rhizomes of turmeric, an Indian spice. Recent studies have also indicated that curcumin has significant benefits for the treatment of cancer and is currently undergoing several clinical trials [1–5]. The anticancer effect of curcumin has been demonstrated in many cell and animal studies [6–11]. Curcumin modulates multiple steps of carcinogenesis and is a feasible therapeutic cancer suppressor. Therefore, for decades, curcumin has attracted increasing interest as an anti-cancer drug.

Curcumin has been shown to possess anticancer activity through different mechanisms, which involve multiple cancer related signalling pathways. Epigenetic alterations correspond to changes in DNA methylation, covalent modifications of histones, or altered miRNA expression patterns. Numerous studies have suggested that curcumin has the potential to target cancer stem cells (CSCs) through the regulation of CSC self-renewal pathways (WNT/β-catenin, Notch, Sonic hedgehog) and specific miRNAs involved in the acquisition of epithelial-mesenchymal transition (EMT) [12]. Recent studies highlighted that curcumin has epigenetic regulatory effects on miRNA in cancers [6, 13–16]. Synergistic effects of curcumin with emodin against the proliferation and invasion of breast cancer cells are through the upregulation of miR-34a [16]. Pharmacological effects of curcumin in lung cancer are mediated by modulation of several miRNAs, such as the downregulation of oncogenic miR-21 and the upregulation of oncosuppressive miR-192-5p and miR-215 [15]. In addition, induction of microRNA-146a is involved in curcumin-mediated enhancement of temozolomide cytotoxicity against human glioblastoma [14].

MicroRNAs (miRNAs) are endogenous, small, non-coding RNAs, approximately 22 nucleotides in length, which typically regulate gene expression at the post-transcriptional level by promoting mRNA degradation or translational repression through binding to complementary sequences in the 3ʹ-untranslated region (3ʹUTR) of target mRNAs [17–20]. miRNAs have been identified as regulators of diverse biological processes, such as cell proliferation, cell cycle, differentiation, organ development, cancer and hormone secretion [21–25]. A growing body of evidence has suggested that miRNAs have crucial roles in different aspects of bone development, osteogenic differentiation, osteoporosis pathophysiology, and osteoclast and osteoblast function. Bone formation is a progression of osteoblast differentiation and maturation. Mesenchymal stem cells (MSCs) can be recruited to bone and then differentiate into osteoblasts, thus promoting this process [26, 27]. Decreased osteogenesis of MSCs contributes to the development of osteoporosis [28]. A series of miRNAs (including miR-133, miR-135, miR-214, miR-216a, miR-497~195 cluster, miR-182, miR-185, miR-219a-5p, miR-940) have been reported to regulate MSC osteogenic differentiation, osteogenic activity, bone formation, and bone loss through targeting of the osteogenic master transcription factors RUNX2 (runt-related transcription factor), OSX (osterix), or regulating osteoblast metabolism-associated pathways [29–35]. The dysregulation of these miRNAs has been linked with skeletal disorders involving a reduction in bone formation [36, 37].

Curcumin can exert its anticancer activity by regulating some miRNAs, while miRNAs also play an important regulatory role in bone formation and osteogenesis. So far, the effects and mechanism of curcumin on bone formation and osteogenesis are relatively unknown and controversial. Whether can curcumin regulate osteogenesis by changing the expression profile of miRNA in MSCs is not clear. In this article, we demonstrate for the first time that curcumin suppresses osteogenesis of (hADSC) through the induction of miR-126a-3p, and subsequently suppresses LRP6 and regulates the WNT pathway. Our study highlights that the osteogenic-suppressing effects of curcumin depend on miR-126a-3p induction and WNT pathway inhibition. Our findings suggest that the use of curcumin as an anti-tumoral agent may lead to decreased bone mass through the suppression of osteogenesis, which might cause a potential threat to tumor metastasis.

## RESULTS

### The effects of curcumin on the regulation of hADSC osteogenesis

**Figure 1:**
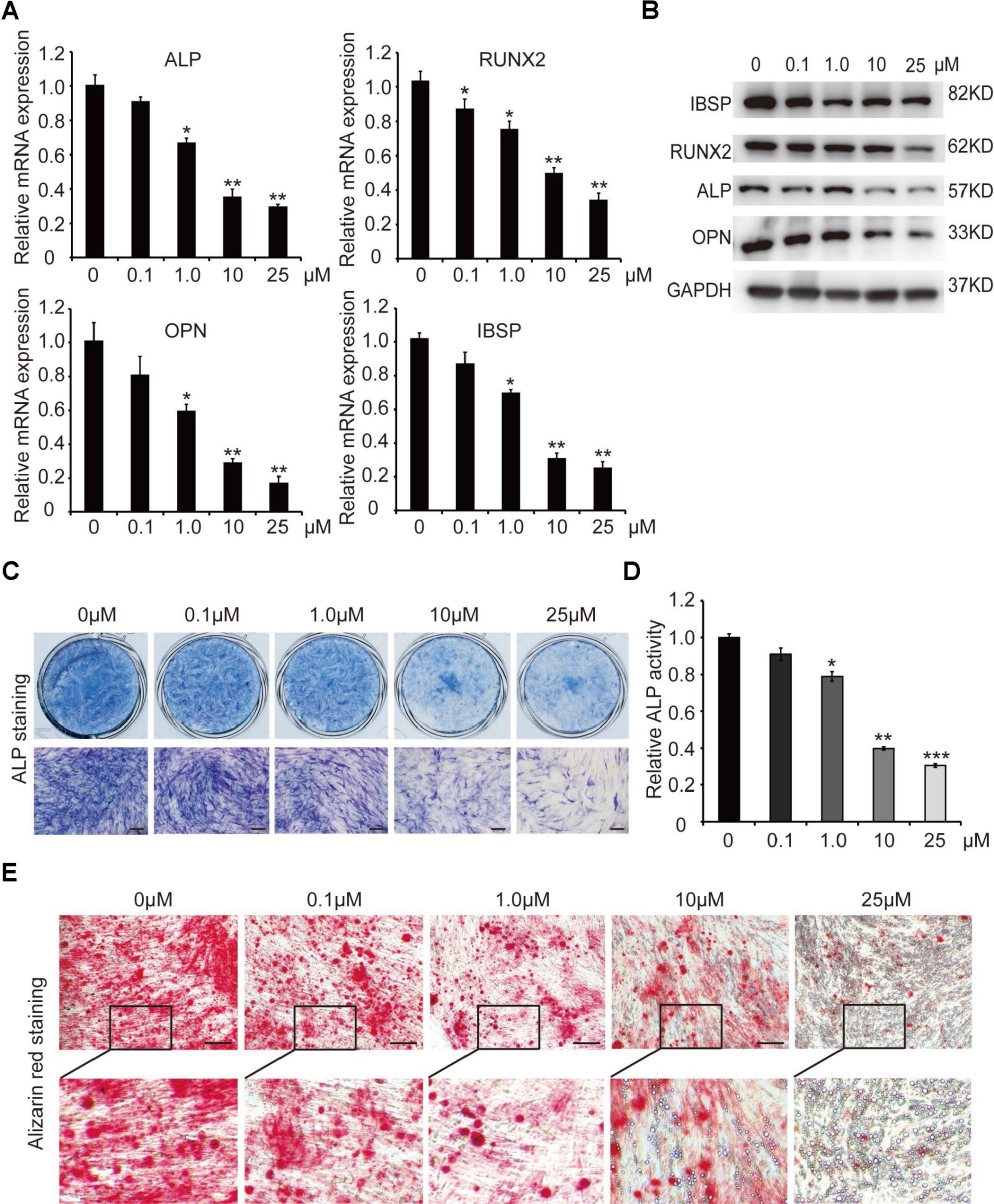
**The effect of different concentrations of curcumin on osteogenic differentiation of hADSCs.** (**A**) qRT-PCR detected the mRNA levels of osteogenic-related genes in curcumin- treated cells on day 6. (**B**) Western blot assays analysed the protein levels osteogenic related genes in curcumin-treated cells. (**C**, **D**) ALP staining and ALP activity analyses indicated early differentiation on day 6. (**E**) Alizarin red staining was performed to detect calcium salt deposits on day 12. Scale bars: 200 μm. Quantitative data are presented as the mean ± S.D. (n =3). *P<0.05; **P<0.01; ***P<0.001. Representative images are shown.

In order to investigate the effect of curcumin (Cur) on osteogenic differentiation of hADSCs, different concentrations (0.1μM, 1.0μM, 10μM, and 25μM) of curcumin were added to the osteoblast induction medium, and hADSCs were induced into osteogenic lineage. qRT-PCR and western blot analyses showed that mRNA and protein levels of key osteogenic factors and the marker genes ALP (alkaline phosphatase), RUNX2, OPN (osteopontin), and IBSP (integrin binding sialoprotein) were sharply decreased in 1.0μM, 10μM, and 25μM curcumin-treated cells, compared with control cells (0 μM curcumin) in the process of osteogenic differentiation (Figure 1A, 1B). The osteoblast phenotype was confirmed by demonstration of repressed ALP staining and ALP activity (Figure 1C, 1D), as well as decreased matrix mineralization detected by alizarin red staining (Figure 1E). These results confirmed that curcumin inhibited osteogenic differentiation of hADSCs in a concentration-dependent manner. Curcumin treatment led to the suppression of osteogenic differentiation at a 1μM concentration, and significant suppression at 10μM and 25μM curcumin treatments. In addition, few calcium salt deposits formed in 10μM-treated cells. Instead, minor lipid droplets formed in 10μM curcumin-treated cells and a large number of lipid droplets formed in 25μM curcumin-treated cells, suggesting that curcumin not only inhibits osteogenesis but also promotes cell differentiation into adipocytes (Figure 1E). For subsequent experiments, curcumin was used at a final concentration of 10μM.

### Curcumin modulates miRNA expression in hADSCs

**Figure 2:**
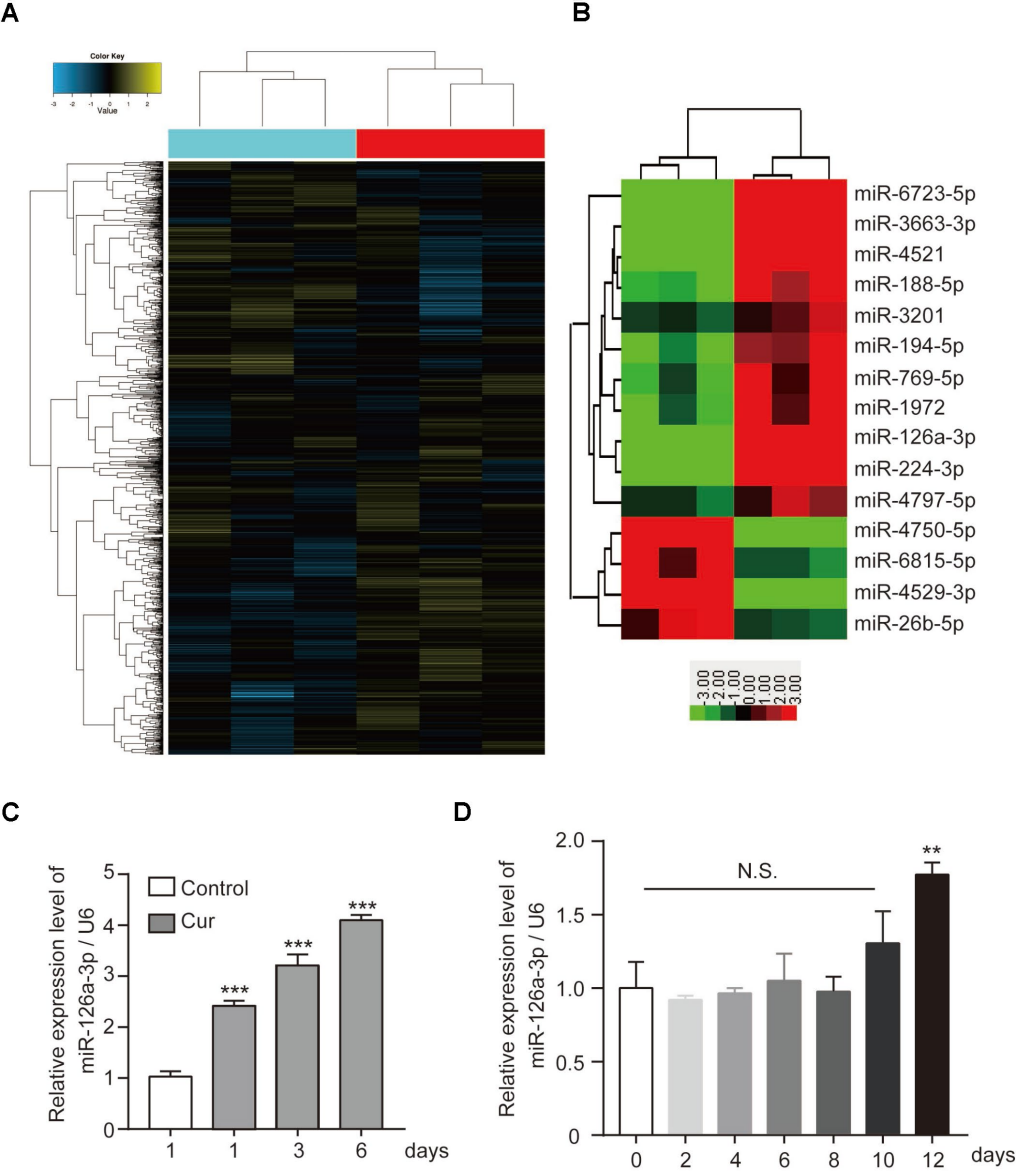
**The effect of curcumin on miRNA expression patterns in hADSCs.** (**A**) Microarray analysis was performed to analyse the miRNA expression pattern in hADSCs. (**B**) miRNAs with at least a 2.5-fold increase or decrease in response to curcumin treatment are shown. (**C**) qRT-PCR detected the expression levels of miR-126a-3p in curcumin-treated hADSCs. (**D**) qRT-PCR detected the expression profile of miR-126a-3p during osteogenic differentiation of hADSCs. Quantitative data are presented as the mean ± S.D. (n =3). *P<0.05; **P<0.01; ***P<0.001.

Recently curcumin was discovered to regulate the development of various tumors by affecting the expression of miRNA. More and more, miRNAs have been found to play an important role in bone development, bone formation, and osteogenesis. Therefore, we hypothesized that curcumin might regulate hADSC osteogenesis by regulating miRNAs. We performed microarray analysis to evaluate if modulation of miRNA expression. Of 1,504 miRNAs detected on the microarray (Figure 2A), we selected by bioinformatics analysis the miRNAs with at least a 2.5-fold increase or decrease in response to curcumin treatment (Figure 2B). Subsequently, we validated the microarray data by qRT-PCR. Among them, the expression level of miR-126a-3p significantly upregulated when curcumin was added to the hADSCs culture medium (Figure 2C), while the qRT-PCR data showed that the endogenous expression level of miR-126a-3p did not change significantly during osteogenic differentiation (Figure 2D), which suggests that miR-126a-3p may be involved in curcumin’s modulation of osteogenesis.

### Overexpression of miR-126a-3p inhibits osteogenic differentiation of hADSCs

**Figure 3:**
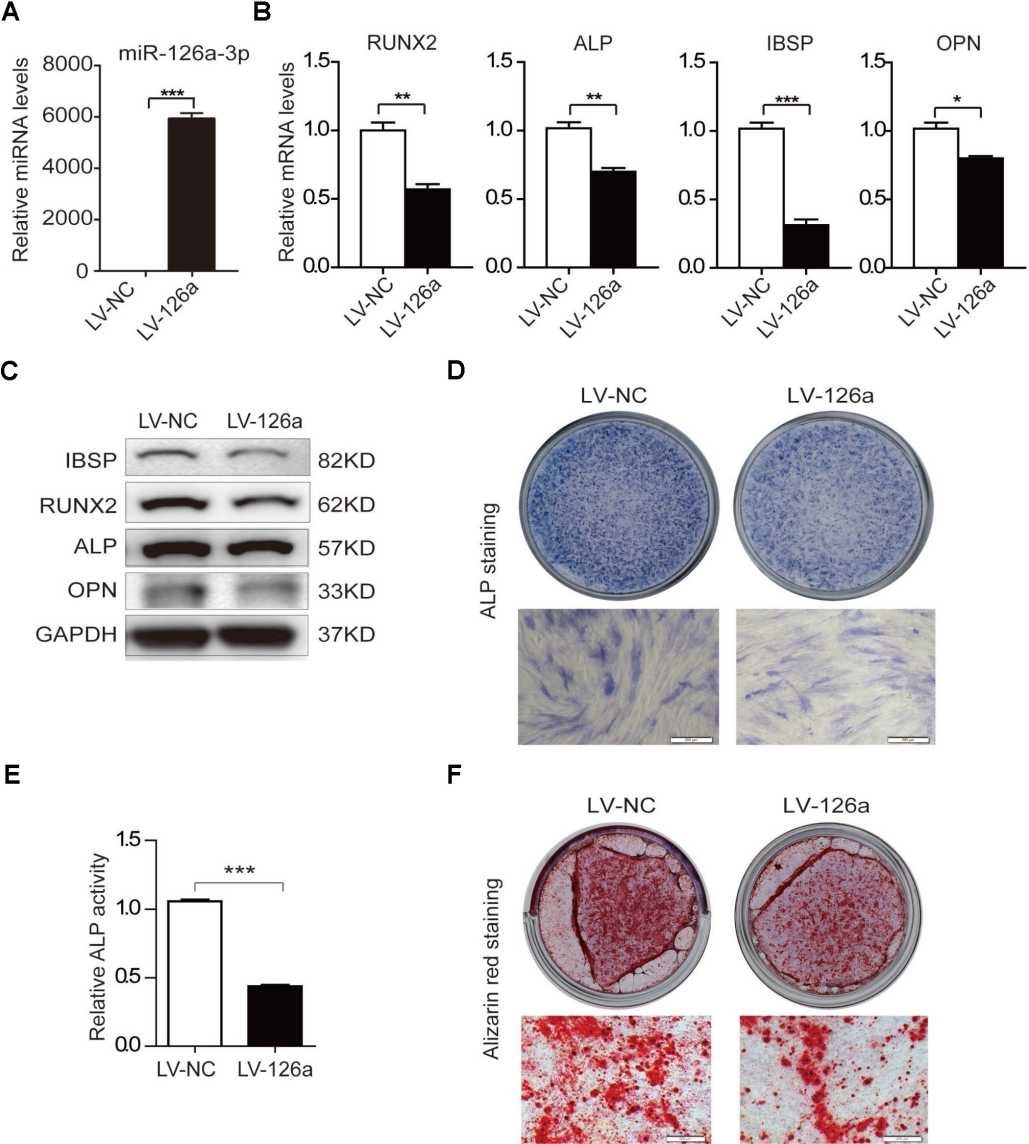
**Osteogenesis is suppressed upon upregulation of miR-126a-3p in hADSCs.** (**A**) The expression level of miR-126a-3p was detected using qRT-PCR in transduced hADSCs with lentivirus overexpressing miR-126a-3p (LV-126a) or the negative control (LV-NC). (**B**) The mRNA levels of osteogenic-related genes were detected by qRT-PCR assay in osteogenic-induced cells on day 6. (**C**) Western blot assays analyzed the protein levels osteogenic-related genes in osteogenic-induced cells. (**D**, **E**) ALP staining and ALP activity analyses indicated early differentiation on day 6 of osteogenic differentiation. (**F**) Alizarin red staining was performed to detect calcium salt deposits on day 12. Scale bars: 200 μm. Quantitative data are presented as the mean ± S.D. (n =3). *P<0.05; **P<0.01; ***P<0.001.

To examine the role of miR-126a-3p in osteogenic differentiation, we transduced hADSCs with a lentivirus overexpressing miR-126a-3p (LV-126a) or the negative control (LV-NC). qRT-PCR analysis confirmed that miR-126a-3p was remarkably upregulated in LV-126a-infected cells (Figure 3A). After induction with osteogenic induction medium for 6 days, we detected the expression of osteogenic transcription factors and marker genes. The qRT-PCR assay revealed that mRNA levels of RUNX2, ALP, IBSP, and OPN were decreased in LV-126a-infected cells compared with those in the LV-NC-infected control cells (Figure 3B). The western blot assay also confirmed that protein levels of IBSP, RUNX2, ALP, and OPN were repressed in LV-126a-infected cells when compared with those in the control cells (Figure 3C). ALP staining and ALP activity indicated that miR-126a-3p overexpression suppressed osteogenic differentiation in hADSCs (Figure 3D, 3E). Alizarin red staining also indicated that matrix mineralization was reduced in LV-126a-infected cells (Figure 3F). These data collectively demonstrated that miR-126a-3p suppresses osteogenic differentiation in hADSCs, which resemble the effects of curcumin.

### Inhibition of miR-126a-3p antagonizes the suppressive effect of curcumin on osteogenic differentiation

**Figure 4:**
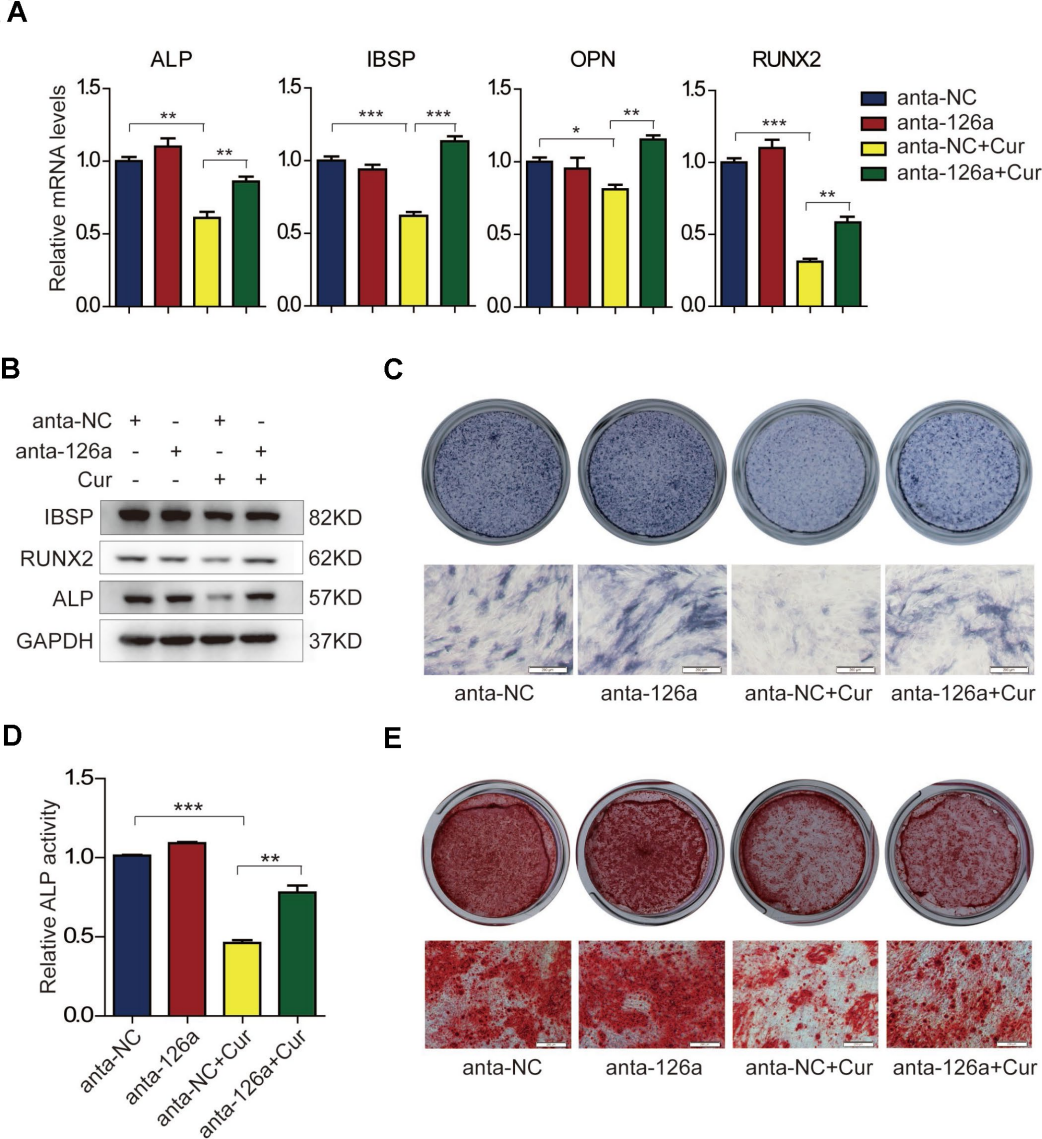
**The suppressive effect of curcumin on osteogenic differentiation is antagonized in miR-126a-3p-inhibited hADSCs.** (**A**) hADSCs were transfected with the miR-126a inhibitor (anta-126a) or the negative control (anta-NC) and were treated with curcumin or untreated. The mRNA levels of osteogenic-related genes were detected by qRT-PCR assay on day 6 of osteogenesis. (**B**) The protein levels of osteogenic-related genes were detected by western blot assay in osteogenic-induced cells. (**C**, **D**) ALP staining, and ALP activity analyses were performed to indicate the early differentiation on day 6 of osteogenic differentiation. (**E**) Alizarin red staining was performed to detect calcium salt deposits on day 12. Scale bars: 200 μm. Quantitative data are presented as the mean ± S.D. (n =3). *P<0.05; **P<0.01; ***P<0.001.

Based on the findings that curcumin and miR-126a-3p could attenuate osteogenic differentiation of hADSCs, and that curcumin treatment could induce the expression of miR-126a-3p, we supposed that miR-126a-3p might play a role in curcumin-mediated inhibition of osteogenic differentiation. To verify this speculation, we blocked the effects of miR-126a-3p using the miR-126a-3p antagomir (anta-126a) and examined the osteogenic differentiation of hADSCs following curcumin exposure. As shown in Figure 4A, curcumin (Cur) treatment dramatically decreased the mRNA levels of osteogenic markers in negative control antagomir (anta-NC)-transfected cells, while transfection of anta-126a significantly elevated the expression levels of the osteogenic markers ALP, IBSP, OPN, and RUNX2 compared with those of the anta-NC-transfected cells under curcumin exposure. Western blot detection of protein levels of ALP, RUNX2, and IBSP also indicated that blocking mR-126a-3p could remarkably restore the expression of osteogenic markers in osteogenic induction medium supplemented with curcumin (Figure 4B). In addition, ALP staining and ALP activity assay (Figure 4C, 4D), as well as mineral deposition detected by alizarin red staining (Figure 4E) consistently revealed that the inhibition of mR-126a-3p rescued the suppressive effect of curcumin on osteogenic differentiation. Together, these data demonstrate that miR-126a-3p is at least in part responsible for the suppression of curcumin of hADSC osteogenic differentiation.

### Human low-density lipoprotein receptor-related protein 6 (LRP6) is a direct target of miR-126a-3p

**Figure 5:**
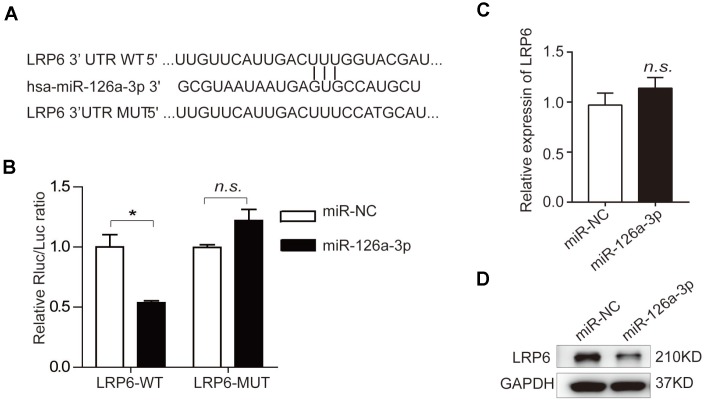
**Prediction and verification of miR-126a-3p target genes.** (**A**) Bioinformatic analysis was used to predict the binding seed sequence of miR-126a-3p with the 3′UTR of LRP6. The wild type (WT) or mutant (MUT) 3′UTR fragments of LRP6 were inserted into the psiCHECK-2 reporter vector. (**B**) The relative luciferase activities were detected using a Dual-Luciferase Reporter Assay System. (**C**, **D**) The mRNA and protein levels of LRP6 were analyzed by qRT-PCR and western blot assays.

To elucidate the mechanisms by which miR-126a-3p-mediated osteogenic regulation, we predicted the potential targets of miR-126a-3p using the bioinformatics tools TargetScan, PicTar, and miRanda. Among the predicted targets, we focused on LRP6 because it possesses a miR-126a-3p binding site in the 3′-UTR region and also plays an important role in osteogenesis [38–40]. To validate whether LRP6 was a bona fide target of miR-126a-3p, we performed a dual luciferase reporter assay by inserting 3′UTR fragments of LRP6 containing the wildtype or a mutant miR-126a-3p binding site into the psiCHECK-2 vector (Figure 5A). Relative luciferase activity (RLA) of the wildtype LRP6 construct was markedly impaired by miR-126a-3p overexpression, while the RLA of the binding site mutant construct was unaffected (Figure 5B). Moreover, miR-126a-3p overexpression significantly downregulated LRP6 at the protein level, but not at the mRNA level in hADSCs (Figure 5C, 5D), suggesting that miR-126a-3p regulates LRP6 expression at the post-transcriptional level. These findings indicated that LRP6 is a direct target of miR-126a-3p in the regulation of osteogenic differentiation.

### Knockdown of endogenous LRP6 resembles the effect of miR-126a-3p and curcumin via the inhibition of the WNT pathway

**Figure 6:**
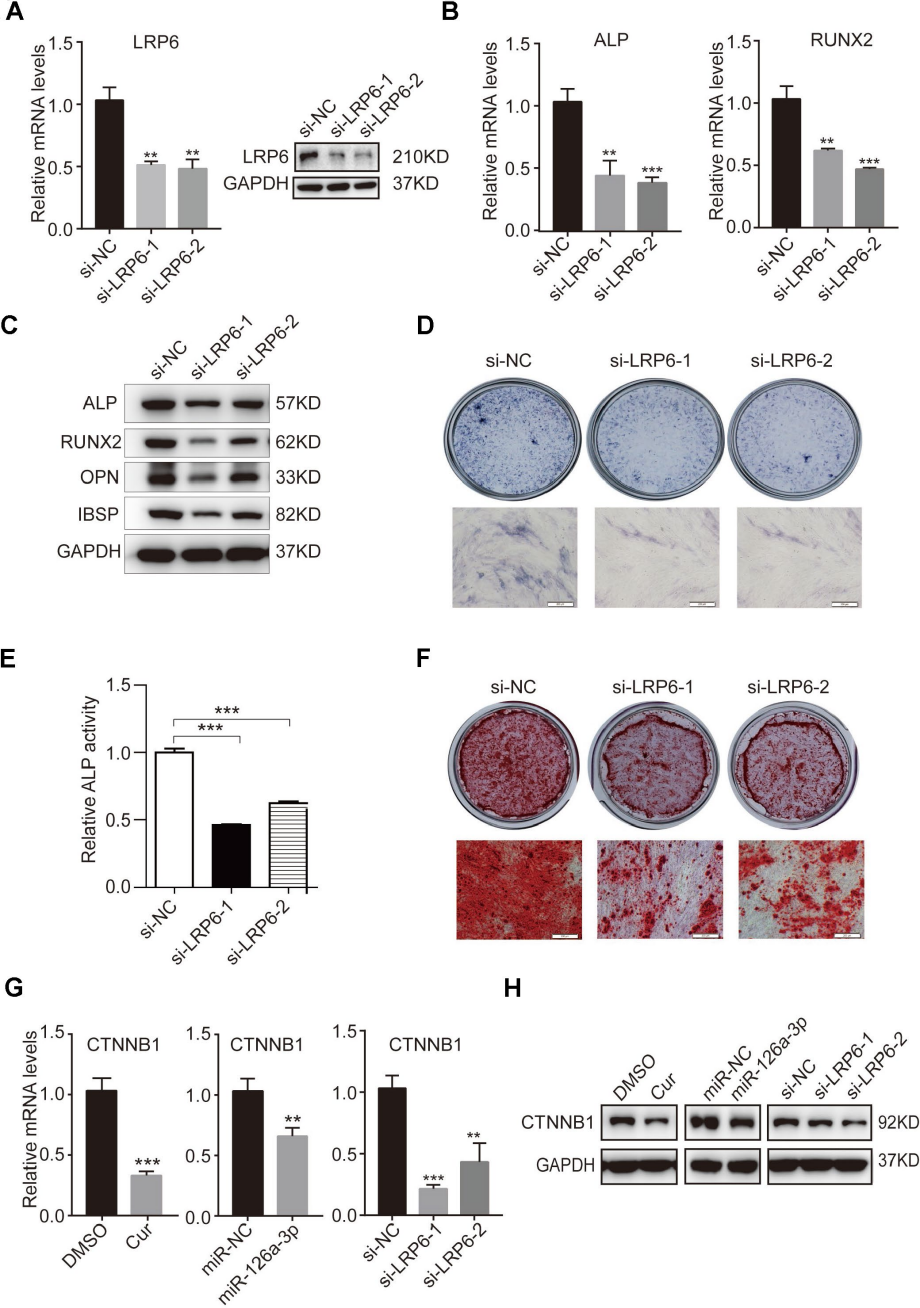
**Knockdown of endogenous LRP6 resembles the effect of miR-126a-3p on osteogenesis via inhibition of the WNT pathway.** (**A**) The mRNA and protein levels of LPR6 were detected by qRT-PCR and western blot assays respectively in LPR6 siRNAs or negative control siRNA-transfected cells. (**B**) The mRNA levels of osteogenic-related genes were detected by qRT-PCR assay on day 6 of osteogenic differentiation. (**C**) The protein levels of osteogenic-related genes were analyzed using western blot assays on day 6 of osteogenic differentiation. (**D**, **E**) ALP staining and ALP activity analyses were used to indicate the early differentiation on day 6 of osteogenic differentiation. (**F**) Alizarin red staining was performed to indicate calcium salt deposits on day 12. (**G**, **H**) qRT-PCR and Western blot assays analyzed the expression levels of CTNNB1. Scale bars: 200 μm. Quantitative data are presented as the mean ± S.D. (n =3). *P<0.05; **P<0.01; ***P<0.001.

To investigate the role of LRP6 in osteogenic differentiation and further confirm that LRP6 is a direct target of miR-126a-3p in the regulation of osteogenesis, we suppressed the expression of LRP6 in hADSCs using two siRNAs (si-LRP6-1 and si-LRP6-2). Knockdown efficiency was confirmed by qRT-PCR and western blotting (Figure 6A) as compared with the negative control (si-NC). Then, we induced hADSCs to differentiate into an osteogenic lineage and detected the expression of osteogenic marker genes at both mRNA and protein levels. After osteogenic induction, the expression of ALP, RUNX2, OPN, and IBSP were significantly decreased in LRP6-depleted cells compared with the control cells (Figure 6B, 6C). Repressed ALP staining and ALP activity (Figure 6D, 6E), as well as reduced mineral deposition detected by alizarin red staining (Figure 6F), indicated that LRP6 knockdown suppresses the osteogenic differentiation of hADSCs. These results were consistent with those observed in miR-126a-3p-overexpression or curcumin-treated hADSCs, suggesting that miR-126a-3p functionally targets LRP6 to regulate hADSC osteogenic differentiation.

The WNT signalling pathway is an important extracellular pathway that has been identified to play an essential role in osteogenesis, bone formation, and osteoporosis. LRP6 is an important member of the LDL receptor family specifically. As the co-receptor for WNT proteins, LRP6 interacts with the lipid on WNT and thereby promotes the WNT/β-catenin (CTNNB1) signalling pathway. Western blot analysis indicated that curcumin treatment inhibits WNT signals in hADSCs. After miR-126a-3p is overexpressed, the suppressive effect on WNT signals in cells is both similar to that of curcumin treatment and endogenous LRP6 downregulation (Figure 6G, 6H). These results further confirmed that curcumin inhibited the activation of WNT signalling by increasing the expression of miR-126a-3p, which directly targets and suppresses LRP6 expression, thus inhibiting osteogenic differentiation.

## DISCUSSION

Curcumin has attracted increasing interest as an anti-cancer drug for decades. Bone metabolism (including bone formation and bone absorption) is closely related to tumour metastasis. However, the effects and mechanism of curcumin’s effects on bone mass is relatively unknown and controversial. We demonstrated that curcumin inhibits osteogenic differentiation of hADSCs in a concentration-dependent manner. Curcumin significantly affects the expression of miRNAs in mesenchymal stem cells. Curcumin upregulates miR-126a-3p expression, miR-126a-3p directly targets and inhibits LRP6, blocking WNT activation, thereby inhibiting osteogenesis of hADSCs.

The anti-tumor effects and mechanism of curcumin have been widely studied, but the role of curcumin on the regulation of bone formation and metabolism is poorly understood. Recently, one study indicated that curcumin could inhibit the osteogenic mimetic properties which occur in castration-resistant prostate cancer cells, by interfering with the common denominators between these cancer cells and bone cells (osteoblasts and osteoclasts) in the metastatic tumor microenvironment [41]. Another report strongly suggests that curcumin modulates TGF-β signalling that occurs due to bone matrix degradation by up-regulating the metastatic-inhibiting bone morphogenic protein-7 (BMP-7) [41]. However, other reports showed that treatment with curcumin attenuates modelled microgravity-induced bone loss [42] and sub-lesional bone loss, following spinal cord injury in rats [43]. Curcumin also alleviates glucocorticoid-induced osteoporosis by protecting osteoblasts from apoptosis [44]. The *in vivo* results of ovariectomy (OVX)-induced osteoporosis model showed that CUR-CGNPs significantly improved bone density and prevented bone loss [45]. Reduced bone mass and reduced bone density are favorable conditions for tumor metastasis. Therefore, it is necessary to clarify the effect of curcumin on bone mass to guide its clinical application better.

Differentiation of mesenchymal stem cells into osteoblasts (named as osteogenic differentiation or osteogenesis) is one of the important factors that determine bone mass. We treated MSCs with different concentrations of curcumin and found that curcumin produced a significant inhibition of osteogenesis, which showed a significant concentration-dependence. Curcumin not only inhibits osteogenesis, but also promotes cell differentiation into adipocytes. In terms of the effect of curcumin on bone formation, the long-term or excessive use of curcumin may lead to osteoporosis or promote tumor progression. Bone homeostasis determines bone mass and is tightly regulated by osteoblasts and osteoclasts. One study reported that osteoclast differentiation was inhibited by gold nanoparticles functionalized with cyclodextrin-curcumin complexes [45]. Another study demonstrated that curcumin inhibited OVX-induced bone loss, at least in part by reducing osteoclastogenesis as a result of increased antioxidant activity and impaired RANKL signalling [46]. Therefore, the effect of curcumin on bone mass requires a comprehensive assessment of the impact of curcumin on bone formation (osteoblast differentiation or osteogenesis) and bone degradation (osteoclast differentiation or osteoclastogenesis).

Increasingly, studies have reported that curcumin can affect the development of a variety of tumors by regulating miRNAs. For instance, the pharmacological effects of curcumin in lung cancer are mediated by the downregulation of oncogenic miR-21 and the upregulation of oncosuppressive miR-192-5p and miR-215 [15]. Curcumin mediates the enhancement of temozolomide cytotoxicity against human glioblastoma via inducing the expression of miR-146a [14]. Curcumin inhibits prostate cancer by upregulating miR-143 [47]. Curcumin exerts its cytotoxic effects against SKOV3 ovarian cancer cells largely through the upregulation of miR-9 and the subsequent modulation of the Akt/FOXO1 axis [48]. Curcumin ameliorates podocyte adhesive capacity damage under mechanical stress by inhibiting miR-124 expression [49].

Many of these miRNAs that can be regulated by curcumin also have been reported to be involved in the regulation of osteogenesis. For instance, miRNA-21 promotes osteogenic differentiation by the PI3K/β-catenin pathway in mesenchymal stem cells [50] and by targeting Smad5 in periodontal ligament stem cells [51]. Dysregulation of miR-146a impairs osteogenesis of bone mesenchymal stem cells under inflammation [52]. MiR-143 suppresses osteogenic differentiation by targeting Osterix [53]. MiR-9 promotes osteoblast differentiation of mesenchymal stem cells by inhibiting DKK1 gene expression [54]. MiR-124 regulates osteoblast differentiation through GSK-3β in ankylosing spondylitis [55]. The expression and function of miRNA have tissue and cell specificity, therefore whether curcumin can also affect osteoblastic differentiation of mesenchymal stem cells by dysregulating the expression of the above miRNAs, or other unknown miRNAs, remains to be further confirmed. To investigate whether curcumin affects the expression profile of miRNA in MSCs and can regulate the differentiation of MSCs to osteoblasts by changing the expression of miRNA, we compared the expression pattern of miRNAs in MSCs before and after curcumin treatment using a microarray. We found that curcumin significantly upregulates the expression of miR-126a-3p in MSCs. Overexpression of miR-126a-3p inhibits the differentiation of MSCs to osteoblasts, and inhibition of miR-126a-3p promotes this process.

Upon treatment of MSCs with curcumin and then inhibiting the expression of miR-126a-3p, the curcumin inhibition of osteogenesis was significantly weakened. So far, there are still relatively few studies on the function of miR-126a-3p. In one study, miR-126a-3p is specifically up-regulated in the process of murine embryo implantation [56]. Mir-126a-3p may play a major role in embryo implantation by regulating Itga11, possibly by impairing cell migration and invasion. Another study found that the expression of miR-126a-3p in plasma and circulating angiogenic cells may be related to type 2 diabetes complications [57]. We first found that miR-126a-3p also plays an important role in osteogenesis of hADSCs. We demonstrated that miR-126a-3p is involved in the inhibition of MSC osteogenesis by curcumin.

The WNT signalling pathway is an important extracellular pathway that plays an essential role in cell growth, differentiation, individual development, migration, genetic stability, apoptosis, self-renewal of stem cells, and maintenance of adult tissue homeostasis [58, 59]. WNT signalling has both pro-cancer and pro-osteogenic activities; dysregulation of WNT signalling is associated with cancers [60] and osteoporosis [61]. Curcumin inhibits cancer cell proliferation in multiple cancer cells and modulates WNT/β-catenin signalling, and apoptotic pathways in A375 cells [62]. The WNT/β-catenin signalling pathway also contributes to the inhibition of hepatocellular carcinoma by curcumin [63]. A missense mutation in LRP6 (as a co-receptor for WNT signalling proteins for the canonical WNT signalling pathway) that resulted in impaired WNT signalling was reported in a family with autosomal dominant early coronary artery disease, metabolic risk factors and osteoporosis [64]. LRP6 heterozygous mice display reduced bone mass [65]. In this study, we found that curcumin inhibits osteogenesis through the upregulated expression of miR-126a-3p. Through bioinformatics prediction and a dual luciferase reporter assay, we found that miR-126a-3p can directly target and inhibit LRP6 expression, then suppress WNT activation, and thus inhibit osteogenesis. Downregulation of endogenous LRP6 can significantly suppress WNT activation and block the osteogenic differentiation of hMSCs, which was similar to that of curcumin treatment or miR-126a-3p overexpression.

Our findings demonstrate that curcumin can suppress osteogenesis of MSCs in a concentration-dependent manner via the upregulation of miR-126a-3p expression, and then miR-126a-3p directly targets and inhibits LRP6 to block WNT activation. Our research suggests that the use of curcumin as an anti-tumor agent may lead to decreased bone mass. Reduced bone mass and reduced bone density are favorable conditions for tumor metastasis. However, bone mass is determined by bone homeostasis, which is tightly regulated by both osteoblasts and osteoclasts. Knowing whether the long-term use or large doses use of curcumin will cause decreased bone mass and bone density, which might lead to a potential risk for tumour metastasis, also requires a neutral assessment of the role of curcumin in both regulating bone formation and bone absorption.

## MATERIALS AND METHODS

### Isolation and expansion of adipose-derived mesenchymal stem cells (hADSCs)

Human adipose tissue was obtained from patients undergoing tumescent liposuction according to procedures approved by the Ethics Committee at the Chinese Academy of Medical Sciences and Peking Union Medical College. hADSCs were isolated and cultured, as previously described [30]. Third-passage hADSCs were used in experiments.

### Osteogenic differentiation of hADSCs

For osteogenic differentiation, hADSCs were seeded into six-well plates until 60-80% confluence and then supplemented with osteogenesis induction medium containing high-glucose DMEM supplemented with 10% FBS, and 10 mM β-glycerophosphate and 0.2 mM ascorbic acid (Sigma-Aldrich, MO, USA). The medium was changed every other day during osteogenic differentiation. To test the effect of curcumin on osteogenic differentiation, 0.1 μM, 1.0 μM, 10 μM or 25 μM curcumin (#C1386, Sigma-Aldrich) was added to the osteogenesis induction medium. The mRNA and protein expression levels of key osteoblast-related genes were analyzed by quantitative real-time PCR and western blotting, respectively. Early osteogenic differentiation was identified by ALPstaining and relative ALP activity assays. Alizarin red staining was used to identify calcium salt deposition.

### ALP staining and the ALP activity assay

ALP staining was performed according to the manufacturer’s instructions (Institute of Haematology and Blood Diseases Hospital, Chinese Academy of Medical Sciences, Tianjin, China). For relative ALP activity assays, cells were washed twice with PBS and lysed in RIPA lysis buffer (Beyotime, Shanghai, China) with 1 mM PMSF. After centrifugation and quantification, 5 μl of protein lysate was incubated with 200 μl of the Alkaline Phosphatase Yellow Liquid Substrate System (pNPP, Sigma-Aldrich) at 37 °C for 30 min. The photometric values were determined using a spectrophotometer at 405 nm and normalized to the protein concentration of cell lysates.

### Alizarin red staining

Alizarin red staining was performed to detect calcium salt deposition on days 12 or 15 after the initiation of osteogenic differentiation. In brief, cells were washed twice with PBS, fixed with 4% paraformaldehyde for 10 min, rinsed with double-distilled H_2_O, and stained with 1% alizarin red (pH 4.2; Leagene, Beijing, China) solution for 30 min at room temperature. Cells were then washed with double-distilled water to remove the unbound dye and were imaged by light microscopy.

### The miRNA mimic and inhibitor, and siRNA transfection

SiRNA used to knockdown LRP mRNA, and the negative control were designed and synthesized by RiboBio Company (Guangzhou, China) (listed in Table 1). For transfection of the miRNA mimic, inhibitor, and siRNAs, Lipofectamine 2000 (Life Technology, USA) was used according to the manufacturer’s recommendations. Lentivirus expressing the miR-126a-3p mimic or inhibitor was constructed and packaged by Genepharma (Suzhou, China). For stable overexpression or inhibition of miR-126a-3p, hADSCs were infected by lentivirus at MOI=10 for 24 h and followed by selection with 1 μg/ml puromycin.

**Table 1:** All primers and siRNAs used in this study.

**Gene**	**Primer sequence**	**Product size (bp)**
LRP6	F: 5′- TGTCAGCGAAGAAGCCATTAAA-3′	231
	R: 5′-TCTAAGGCAATAGCTCTGGGT -3′	
RUNX2	F: 5′-TGTCATGGCGGGTAACGAT-3′	147
	R: 5′-AAGACGGTTATGGTCAAGGTGAA-3′	
ALP	F: 5′-CCACGTCTTCACATTTGGTG-3′	196
	R: 5′-AGACTGCGCCTGGTAGTTGT-3′	
OPN	F: 5′-ACTCGAACGACTCTGATGATGT-3′	224
	R: 5′-GTCAGGTCTGCGAAACTTCTTA-3′	
OC	F: 5′-GGCAGCGAGGTAGTGAAGA-3′	148
	R: 5′-CCTGAAAGCCGATGTGGT-3′	
COL1A1	F: 5′-CCCAAGGAAAAGAAGCACGTC-3′	109
	R: 5′-AGGTCAGCTGGATAGCGACATC-3′	
IBSP	F: 5′-CCCCACCTTTTGGGAAAACCA-3′	109
	R: 5′-TCCCCGTTCTCACTTTCATAGAT-3′	
GAPDH	F: 5′-GGTCACCAGGGCTGCTTTTA-3′	195
	R: 5′-GGATCTCGCTCCTGGAAGATG-3′	
CTNNB1	F: 5′-CAGAGTGCTGAAGGTGCTATC-3′	177
	R: 5′-CCTTCCATCCCTTCCTGTTTAG-3′	
NC	r(UUCUCCGAACGUGUCACGU)dTdT	
si-LRP6-1	r(GCTCAACCGTGAAGTTATA)dTdT	
si-LRP6-2	r(GGGAAACTATGACTAATGA)dTdT	

### RNA extraction and quantitative real-time PCR (qRT-PCR) analysis

Total RNA was extracted using TRIzol reagent (Invitrogen, Carlsbad, CA, USA) and was treated with DNase I (Ambion, USA) in accordance with the manufacturer’s instructions. Reverse transcription and qRT-PCR were performed as previously described [30]. Relative expression levels of mRNA or miRNA were evaluated using the 2^-ΔΔCt^ method and were normalized to the expression of GAPDH or U6, respectively. Primer sequences are shown in Table 1.

### MiRNA microarray analysis

MiRNA expression profiling was performed using the TaqMan Array Human MicroRNA Cards V3.0 (Applied Biosystems, Foster City, CA, P/N: 4444913) following the manufacturer’s instructions. Ten nanograms of total RNA were used for each reaction. The expression of each miRNA was plotted as the average CT value for each sample minus the average value for U6 using the 2^−ΔΔCT^ method. Experiments were performed in triplicate. Clustering of data and heat-map representations were performed using Cluster 3.0 and Treeview software.

### Western blot analysis

Western blotting was performed as we previously described [30]. Antibodies against ALP (ab108337) and OPN (ab69498) were purchased from Abcam (Cambridge, MA, USA). Antibodies against IBSP (5468) and RUNX2 (8486) were purchased from Cell Signaling Technology (Danvers, MA, USA). Antibodies against GAPDH (10494-1-AP) and β-actin (HRP-60008) were purchased from Proteintech (Wuhan, China).

### Dual luciferase reporter assay

The wild type 3′UTR of LRP6 was obtained by PCR and inserted into the luciferase reporter vector psiCHECK2 (Promega, Madison, WI, USA) between XhoI and Not I sites. The mutant was made by replacing the predicted binding sequences of miR-126a-3p with scrambled sequences. The 293T cells were seeded in 24-well plates and cotransfected with 40 nmol/L of miRNAs (NC or miR-126a-3p mimics) and 50 ng of psiCHECK2 (wildtype or mutant) using Lipofectamine 2000. *Renilla* and firefly luciferase activity were measured using the Dual-Luciferase® Reporter Assay System (#E1910, Promega) 24 h after transfection.

### Statistical analysis

The data from at least three independent samples or repeated experiments were analysed using GraphPad Prism5 software (GraphPad Software Inc.) and presented as the mean ± SD. Data between two groups were assessed using two-tailed Student’s *t*-tests. Differences were considered statistically significant at *P<0.05, **P<0.01 and ***P<0.001.

## References

[1] Su P, Yang Y, Wang G, Chen X, Ju Y. Curcumin attenuates resistance to irinotecan via induction of apoptosis of cancer stem cells in chemoresistant colon cancer cells. Int J Oncol. 2018; 53:1343–53. 10.3892/ijo.2018.4461 29956726

[2] Sharma RA, Euden SA, Platton SL, Cooke DN, Shafayat A, Hewitt HR, Marczylo TH, Morgan B, Hemingway D, Plummer SM, Pirmohamed M, Gescher AJ, Steward WP. Phase I clinical trial of oral curcumin: biomarkers of systemic activity and compliance. Clin Cancer Res. 2004; 10:6847–6854. 10.1158/1078-0432.CCR-04-0744 15501961

[3] Ryan JL, Heckler CE, Ling M, Katz A, Williams JP, Pentland AP, Morrow GR. Curcumin for radiation dermatitis: a randomized, double-blind, placebo-controlled clinical trial of thirty breast cancer patients. Radiat Res. 2013; 180:34–43. 10.1667/RR3255.1 23745991 PMC3998827

[4] Jalili-Nik M, Soltani A, Moussavi S, Ghayour-Mobarhan M, Ferns GA, Hassanian SM, Avan A. Current status and future prospective of Curcumin as a potential therapeutic agent in the treatment of colorectal cancer. J Cell Physiol. 2018; 233:6337–45. 10.1002/jcp.26368 29219177 PMC13482484

[5] Tan BL, Norhaizan ME. Curcumin Combination Chemotherapy: The Implication and Efficacy in Cancer. Molecules. 2019; 24:E2527. 10.3390/molecules24142527 31295906 PMC6680685

[6] Zhu M, Zheng Z, Huang J, Ma X, Huang C, Wu R, Li X, Liang Z, Deng F, Wu J, Geng S, Xie C, Zhong C. Modulation of miR-34a in curcumin-induced antiproliferation of prostate cancer cells. J Cell Biochem. 2019; 120:15616–24. 10.1002/jcb.28828 31042325

[7] Mapoung S, Suzuki S, Fuji S, Naiki-Ito A, Kato H, Yodkeeree S, Ovatlarnporn C, Takahashi S, Limtrakul Dejkriengkraikul P. Cyclohexanone curcumin analogs inhibit the progression of castration-resistant prostate cancer in vitro and in vivo. Cancer Sci. 2019; 110:596–607. 10.1111/cas.13897 30499149 PMC6361605

[8] Ma L, Zhang X, Wang Z, Huang L, Meng F, Hu L, Chen Y, Wei J. Anti-cancer effects of curcumin on myelodysplastic syndrome through the inhibition of enhancer of zeste homolog-2 (EZH2). Curr Cancer Drug Targets. 2019. [Epub ahead of print]. 10.2174/1568009619666190212121735 30747066

[9] Kuttikrishnan S, Siveen KS, Prabhu KS, Khan AQ, Ahmed EI, Akhtar S, Ali TA, Merhi M, Dermime S, Steinhoff M, Uddin S. Curcumin Induces Apoptotic Cell Death via Inhibition of PI3-Kinase/AKT Pathway in B-Precursor Acute Lymphoblastic Leukemia. Front Oncol. 2019; 9:484. 10.3389/fonc.2019.00484 31275848 PMC6593070

[10] Karimpour M, Feizi MAH, Mahdavi M, Krammer B, Verwanger T, Najafi F, Babaei E. Development of curcumin-loaded gemini surfactant nanoparticles: Synthesis, characterization and evaluation of anticancer activity against human breast cancer cell lines. Phytomedicine. 2019; 57:183–190. 10.1016/j.phymed.2018.11.017 30776589

[11] El-Far M, Salah N, Essam A, Abd El-Azim A, Karam M, El-Sherbiny IM. Potential anticancer activity and mechanism of action of nanoformulated curcumin in experimental Ehrlich ascites carcinoma-bearing animals. Nanomedicine (Lond). 2019; 14:553–73. 10.2217/nnm-2018-0298 30810086

[12] Li Y, Zhang T. Targeting cancer stem cells by curcumin and clinical applications. Cancer Lett. 2014; 346:197–205. 10.1016/j.canlet.2014.01.012 24463298

[13] Zhou S, Li J, Xu H, Zhang S, Chen X, Chen W, Yang S, Zhong S, Zhao J, Tang J. Liposomal curcumin alters chemosensitivity of breast cancer cells to Adriamycin via regulating microRNA expression. Gene. 2017; 622:1–12. 10.1016/j.gene.2017.04.026 28431975

[14] Wu H, Liu Q, Cai T, Chen YD, Wang ZF. Induction of microRNA-146a is involved in curcumin-mediated enhancement of temozolomide cytotoxicity against human glioblastoma. Mol Med Rep. 2015; 12:5461–66. 10.3892/mmr.2015.4087 26239619

[15] Lelli D, Pedone C, Majeed M, Sahebkar A. Curcumin and Lung Cancer: the Role of microRNAs. Curr Pharm Des. 2017; 23:3440–44. 10.2174/1381612823666170109144818 28067164

[16] Guo J, Li W, Shi H, Xie X, Li L, Tang H, Wu M, Kong Y, Yang L, Gao J, Liu P, Wei W, Xie X. Synergistic effects of curcumin with emodin against the proliferation and invasion of breast cancer cells through upregulation of miR-34a. Mol Cell Biochem. 2013; 382:103–11. 10.1007/s11010-013-1723-6 23771315

[17] Bartel DP. MicroRNAs: genomics, biogenesis, mechanism, and function. Cell. 2004; 116:281–97. 10.1016/S0092-8674(04)00045-5 14744438

[18] Carthew RW, Sontheimer EJ. Origins and Mechanisms of miRNAs and siRNAs. Cell. 2009; 136:642–55. 10.1016/j.cell.2009.01.035 19239886 PMC2675692

[19] Filipowicz W, Bhattacharyya SN, Sonenberg N. Mechanisms of post-transcriptional regulation by microRNAs: are the answers in sight? Nat Rev Genet. 2008; 9:102–14. 10.1038/nrg2290 18197166

[20] Huntzinger E, Izaurralde E. Gene silencing by microRNAs: contributions of translational repression and mRNA decay. Nat Rev Genet. 2011; 12:99–110. 10.1038/nrg2936 21245828

[21] Yang M, Liu R, Li X, Liao J, Pu Y, Pan E, Yin L, Wang Y. miRNA-183 suppresses apoptosis and promotes proliferation in esophageal cancer by targeting PDCD4. Mol Cells. 2014; 37:873–80. 10.14348/molcells.2014.0147 25518924 PMC4275704

[22] Ventura A, Young AG, Winslow MM, Lintault L, Meissner A, Erkeland SJ, Newman J, Bronson RT, Crowley D, Stone JR, Jaenisch R, Sharp PA, Jacks T. Targeted deletion reveals essential and overlapping functions of the miR-17 through 92 family of miRNA clusters. Cell. 2008; 132:875–86. 10.1016/j.cell.2008.02.019 18329372 PMC2323338

[23] Poy MN, Eliasson L, Krutzfeldt J, Kuwajima S, Ma X, Macdonald PE, Pfeffer S, Tuschl T, Rajewsky N, Rorsman P, Stoffel M. A pancreatic islet-specific microRNA regulates insulin secretion. Nature. 2004; 432:226–30. 10.1038/nature03076 15538371

[24] Mori M, Triboulet R, Mohseni M, Schlegelmilch K, Shrestha K, Camargo FD, Gregory RI. Hippo signaling regulates microprocessor and links cell-density-dependent miRNA biogenesis to cancer. Cell. 2014; 156:893–906. 10.1016/j.cell.2013.12.043 24581491 PMC3982296

[25] Brennecke J, Hipfner DR, Stark A, Russell RB, Cohen SM. bantam encodes a developmentally regulated microRNA that controls cell proliferation and regulates the proapoptotic gene hid in Drosophila. Cell. 2003; 113:25–36. 10.1016/S0092-8674(03)00231-9 12679032

[26] Wang Z, Wang D, Yang D, Zhen W, Zhang J, Peng S. The effect of icariin on bone metabolism and its potential clinical application. Osteoporos Int. 2018; 29:535–544. 10.1007/s00198-017-4255-1 29110063

[27] Crisan M, Yap S, Casteilla L, Chen CW, Corselli M, Park TS, Andriolo G, Sun B, Zheng B, Zhang L, Norotte C, Teng PN, Traas J, et al. A perivascular origin for mesenchymal stem cells in multiple human organs. Cell Stem Cell. 2008; 3:301–13. 10.1016/j.stem.2008.07.003 18786417

[28] Qi M, Zhang L, Ma Y, Shuai Y, Li L, Luo K, Liu W, Jin Y. Autophagy Maintains the Function of Bone Marrow Mesenchymal Stem Cells to Prevent Estrogen Deficiency-Induced Osteoporosis. Theranostics. 2017; 7:4498–516. 10.7150/thno.17949 29158841 PMC5695145

[29] Wang X, Guo B, Li Q, Peng J, Yang Z, Wang A, Li D, Hou Z, Lv K, Kan G, Cao H, Wu H, Song J, et al. miR-214 targets ATF4 to inhibit bone formation. Nat Med. 2013; 19:93–100. 10.1038/nm.3026 23223004

[30] Li H, Li T, Fan J, Li T, Fan L, Wang S, Weng X, Han Q, Zhao RC. miR-216a rescues dexamethasone suppression of osteogenesis, promotes osteoblast differentiation and enhances bone formation, by regulating c-Cbl-mediated PI3K/AKT pathway. Cell Death Differ. 2015; 22:1935–45. 10.1038/cdd.2015.99 26206089 PMC4816120

[31] Inoue K, Deng Z, Chen Y, Giannopoulou E, Xu R, Gong S, Greenblatt MB, Mangala LS, Lopez-Berestein G, Kirsch DG, Sood AK, Zhao L, Zhao B. Bone protection by inhibition of microRNA-182. Nat Commun. 2018; 9:4108. 10.1038/s41467-018-06446-0 30291236 PMC6173760

[32] Hashimoto K, Ochi H, Sunamura S, Kosaka N, Mabuchi Y, Fukuda T, Yao K, Kanda H, Ae K, Okawa A, Akazawa C, Ochiya T, Futakuchi M, et al. Cancer-secreted hsa-miR-940 induces an osteoblastic phenotype in the bone metastatic microenvironment via targeting ARHGAP1 and FAM134A. Proc Natl Acad Sci USA. 2018; 115:2204–09. 10.1073/pnas.1717363115 29440427 PMC5834702

[33] Grunhagen J, Bhushan R, Degenkolbe E, Jager M, Knaus P, Mundlos S, Robinson PN, Ott CE. MiR-497∼195 cluster microRNAs regulate osteoblast differentiation by targeting BMP signaling. J Bone Miner Res. 2015; 30:796–808. 10.1002/jbmr.2412 25407900

[34] Cui Q, Xing J, Yu M, Wang Y, Xu J, Gu Y, Nan X, Ma W, Liu H, Zhao H. Mmu-miR-185 depletion promotes osteogenic differentiation and suppresses bone loss in osteoporosis through the Bgn-mediated BMP/Smad pathway. Cell Death Dis. 2019; 10:172. 10.1038/s41419-019-1428-1 30787286 PMC6382812

[35] Aquino-Martinez R, Farr JN, Weivoda MM, Negley BA, Onken JL, Thicke BS, Fulcer MM, Fraser DG, van Wijnen AJ, Khosla S, Monroe DG. miR-219a-5p Regulates Rorbeta During Osteoblast Differentiation and in Age-related Bone Loss. J Bone Miner Res. 2019; 34:135–144. 10.1002/jbmr.3586 30321475 PMC6450079

[36] van der Eerden BC. MicroRNAs in the skeleton: cell-restricted or potent intercellular communicators? Arch Biochem Biophys. 2014; 561:46–55. 10.1016/j.abb.2014.04.016 24832391

[37] Lian JB, Stein GS, van Wijnen AJ, Stein JL, Hassan MQ, Gaur T, Zhang Y. MicroRNA control of bone formation and homeostasis. Nat Rev Endocrinol. 2012; 8:212–27. 10.1038/nrendo.2011.234 22290358 PMC3589914

[38] Westendorf JJ, Kahler RA, Schroeder TM. Wnt signaling in osteoblasts and bone diseases. Gene. 2004; 341:19–39. 10.1016/j.gene.2004.06.044 15474285

[39] Li C, Williams BO, Cao X, Wan M. LRP6 in mesenchymal stem cells is required for bone formation during bone growth and bone remodeling. Bone Res. 2014; 2:14006. 10.1038/boneres.2014.6 26273519 PMC4472141

[40] Joeng KS, Schumacher CA, Zylstra-Diegel CR, Long F, Williams BO. Lrp5 and Lrp6 redundantly control skeletal development in the mouse embryo. Dev Biol. 2011; 359:222–29. 10.1016/j.ydbio.2011.08.020 21924256 PMC3220949

[41] Dorai T, Diouri J, O’Shea O, Doty SB. Curcumin Inhibits Prostate Cancer Bone Metastasis by Up-Regulating Bone Morphogenic Protein-7 *in Vivo*. J Cancer Ther. 2014; 5:369–86. 10.4236/jct.2014.54044 24949215 PMC4060744

[42] Xin M, Yang Y, Zhang D, Wang J, Chen S, Zhou D. Attenuation of hind-limb suspension-induced bone loss by curcumin is associated with reduced oxidative stress and increased vitamin D receptor expression. Osteoporos Int. 2015; 26:2665–2676. 10.1007/s00198-015-3153-7 25963235

[43] Yang X, He B, Liu P, Yan L, Yang M, Li D. Treatment with curcumin alleviates sublesional bone loss following spinal cord injury in rats. Eur J Pharmacol. 2015; 765:209–16. 10.1016/j.ejphar.2015.08.036 26300394

[44] Chen Z, Xue J, Shen T, Ba G, Yu D, Fu Q. Curcumin alleviates glucocorticoid-induced osteoporosis by protecting osteoblasts from apoptosis in vivo and in vitro. Clin Exp Pharmacol Physiol. 2016; 43:268–76. 10.1111/1440-1681.12513 26515751

[45] Heo DN, Ko WK, Moon HJ, Kim HJ, Lee SJ, Lee JB, Bae MS, Yi JK, Hwang YS, Bang JB, Kim EC, Do SH, Kwon IK. Inhibition of osteoclast differentiation by gold nanoparticles functionalized with cyclodextrin curcumin complexes. ACS Nano. 2014; 8:12049–62. 10.1021/nn504329u 25420230

[46] Kim WK, Ke K, Sul OJ, Kim HJ, Kim SH, Lee MH, Kim HJ, Kim SY, Chung HT, Choi HS. Curcumin protects against ovariectomy-induced bone loss and decreases osteoclastogenesis. J Cell Biochem. 2011; 112:3159–66. 10.1002/jcb.23242 21732406

[47] Cao H, Yu H, Feng Y, Chen L, Liang F. Curcumin inhibits prostate cancer by targeting PGK1 in the FOXD3/miR-143 axis. Cancer Chemother Pharmacol. 2017; 79:985–94. 10.1007/s00280-017-3301-1 28391351

[48] Zhao SF, Zhang X, Zhang XJ, Shi XQ, Yu ZJ, Kan QC. Induction of microRNA-9 mediates cytotoxicity of curcumin against SKOV3 ovarian cancer cells. Asian Pac J Cancer Prev. 2014; 15:3363–68. 10.7314/APJCP.2014.15.8.3363 24870723

[49] Li D, Lu Z, Jia J, Zheng Z, Lin S. Curcumin ameliorates Podocytic adhesive capacity damage under mechanical stress by inhibiting miR-124 expression. Kidney Blood Press Res. 2013; 38:61–71. 10.1159/000355755 24556741

[50] Meng YB, Li X, Li ZY, Zhao J, Yuan XB, Ren Y, Cui ZD, Liu YD, Yang XJ. microRNA-21 promotes osteogenic differentiation of mesenchymal stem cells by the PI3K/beta-catenin pathway. J Orthop Res. 2015; 33:957–964. 10.1002/jor.22884 25728838

[51] Wei F, Yang S, Guo Q, Zhang X, Ren D, Lv T, Xu X. MicroRNA-21 regulates Osteogenic Differentiation of Periodontal Ligament Stem Cells by targeting Smad5. Sci Rep. 2017; 7:16608. 10.1038/s41598-017-16720-8 29192241 PMC5709498

[52] Kuang W, Zheng L, Xu X, Lin Y, Lin J, Wu J, Tan J. Dysregulation of the miR-146a-Smad4 axis impairs osteogenesis of bone mesenchymal stem cells under inflammation. Bone Res. 2017; 5:17037. 10.1038/boneres.2017.37 29167750 PMC5698258

[53] Li E, Zhang J, Yuan T, Ma B. MiR-143 suppresses osteogenic differentiation by targeting Osterix. Mol Cell Biochem. 2014; 390:69–74. 10.1007/s11010-013-1957-3 24381059

[54] Liu X, Xu H, Kou J, Wang Q, Zheng X, Yu T. MiR-9 promotes osteoblast differentiation of mesenchymal stem cells by inhibiting DKK1 gene expression. Mol Biol Rep. 2016; 43:939–46. 10.1007/s11033-016-4030-y 27393149

[55] Gershy-Damet GM, Koffi KJ. [Utilization of an ELISA technic for the quantification of antipoliovirus antibodies in human sera]. Bull Soc Pathol Exot Filiales. 1987; 80:289–94. 3040283

[56] Li Z, Jia J, Gou J, Tong A, Liu X, Zhao X, Yi T. Mmu-miR-126a-3p plays a role in murine embryo implantation by regulating Itga11. Reprod Biomed Online. 2015; 31:384–93. 10.1016/j.rbmo.2015.05.016 26194885

[57] Olivieri F, Spazzafumo L, Bonafè M, Recchioni R, Prattichizzo F, Marcheselli F, Micolucci L, Mensà E, Giuliani A, Santini G, Gobbi M, Lazzarini R, Boemi M, et al. MiR-21-5p and miR-126a-3p levels in plasma and circulating angiogenic cells: relationship with type 2 diabetes complications. Oncotarget. 2015; 6:35372–82. 10.18632/oncotarget.6164 26498351 PMC4742111

[58] Peng Y, Zhang X, Feng X, Fan X, Jin Z. The crosstalk between microRNAs and the Wnt/β-catenin signaling pathway in cancer. Oncotarget. 2017; 8:14089–106. 10.18632/oncotarget.12923 27793042 PMC5355165

[59] Nusse R, Clevers H. Wnt/β-Catenin Signaling, Disease, and Emerging Therapeutic Modalities. Cell. 2017; 169:985–99. 10.1016/j.cell.2017.05.016 28575679

[60] White BD, Chien AJ, Dawson DW. Dysregulation of Wnt/β-catenin signaling in gastrointestinal cancers. Gastroenterology. 2012; 142:219–32. 10.1053/j.gastro.2011.12.001 22155636 PMC3285553

[61] Kim W, Kim M, Jho EH. Wnt/β-catenin signalling: from plasma membrane to nucleus. Biochem J. 2013; 450:9–21. 10.1042/BJ20121284 23343194

[62] Srivastava NS, Srivastava RAK. Curcumin and quercetin synergistically inhibit cancer cell proliferation in multiple cancer cells and modulate Wnt/beta-catenin signaling and apoptotic pathways in A375 cells. Phytomedicine. 2019; 52:117–128. 10.1016/j.phymed.2018.09.224 30599890

[63] Hu P, Ke C, Guo X, Ren P, Tong Y, Luo S, He Y, Wei Z, Cheng B, Li R, Luo J, Meng Z. Both glypican-3/Wnt/beta-catenin signaling pathway and autophagy contributed to the inhibitory effect of curcumin on hepatocellular carcinoma. Dig Liver Dis. 2019; 51:120–126. 10.1016/j.dld.2018.06.012 30001951

[64] Mani A, Radhakrishnan J, Wang H, Mani A, Mani MA, Nelson-Williams C, Carew KS, Mane S, Najmabadi H, Wu D, Lifton RP. LRP6 mutation in a family with early coronary disease and metabolic risk factors. Science. 2007; 315:1278–82. 10.1126/science.1136370 17332414 PMC2945222

[65] Holmen SL, Giambernardi TA, Zylstra CR, Buckner-Berghuis BD, Resau JH, Hess JF, Glatt V, Bouxsein ML, Ai M, Warman ML, Williams BO. Decreased BMD and limb deformities in mice carrying mutations in both Lrp5 and Lrp6. J Bone Miner Res. 2004; 19:2033–2040. 10.1359/jbmr.040907 15537447

