# Correction for: Curcumin suppresses osteogenesis by inducing miR-126a-3p and subsequently suppressing the WNT/LRP6 pathway

**DOI:** 10.18632/aging.202920

**Published:** 2021-03-31

**Authors:** Hongling Li, Lifeng Yue, Haoying Xu, Na Li, Jing Li, Zhiguo Zhang, Robert Chunhua Zhao

**Affiliations:** 1 Institute of Basic Medical Sciences Chinese Academy of Medical Sciences, School of Basic Medicine Peking Union Medical College, Peking Union Medical College Hospital, Beijing Key Laboratory of New Drug Development and Clinical Trial of Stem Cell Therapy, Beijing, 100005, China; 2 Beijing Dongzhimen Hospital, Beijing University of Chinese Medicine, Beijing, 100700, China; 3 Institute of Basic Theory, China Academy of Chinese Medical Sciences, Beijing, 100700, China

**Keywords:** correction

Original article: Aging. 2019; 11:6983–6998.  . https://doi.org/10.18632/aging.102232

**This article has been corrected:** In Figure 6D authors mistakenly used the same picture for si-LRP6-1 and si-LRP6-2. In the new Figure 6, provided below, they replaced panel 6D. New panel 6D contains new image for si-LRP6-2 from the original set of experiments. The authors guarantee that the correction doesn't affect the interpretation of the data or the conclusions drawn from it.

**Figure 6:**
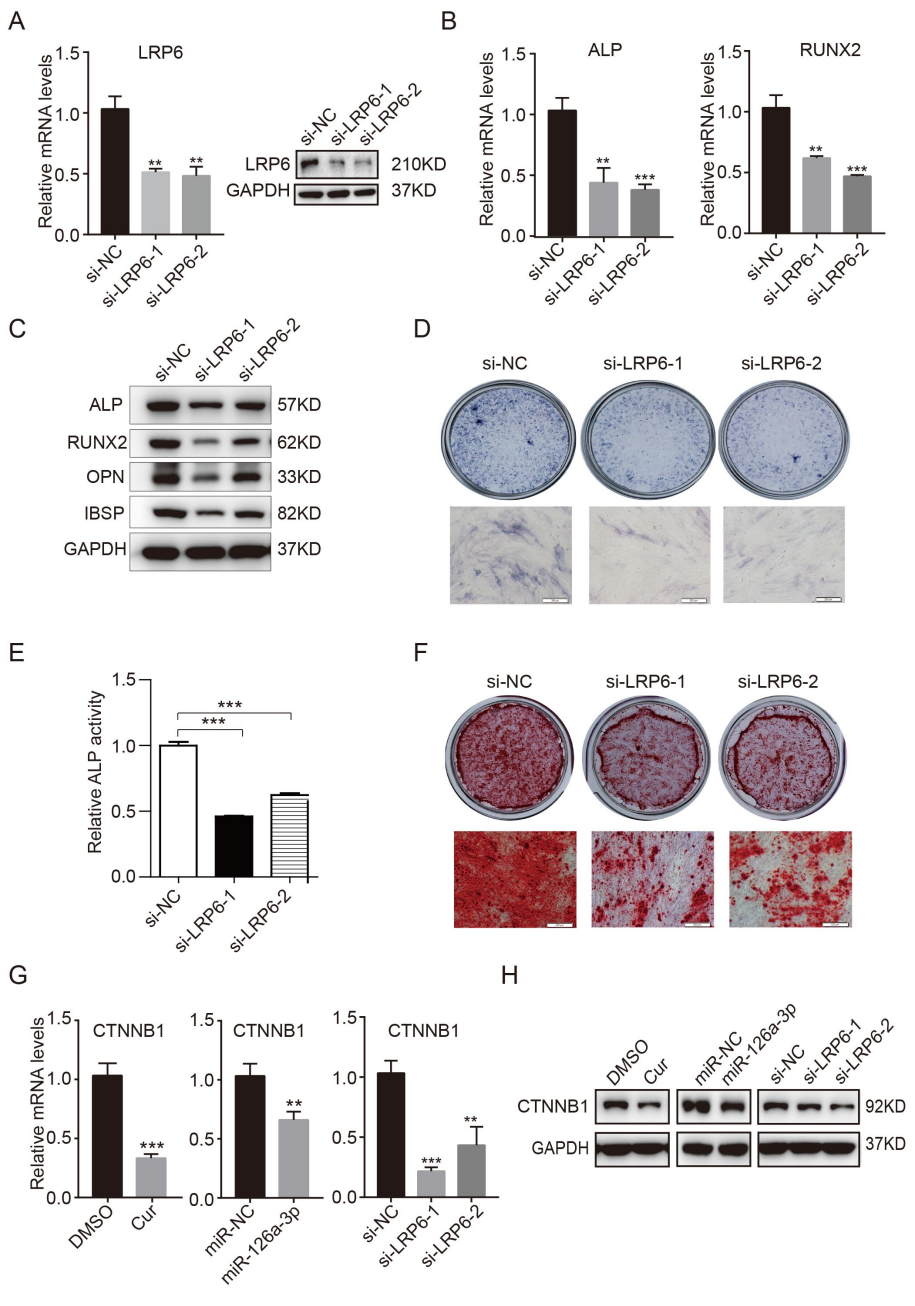
**Knockdown of endogenous LRP6 resembles the effect of miR-126a-3p on osteogenesis via inhibition of the WNT pathway.** (**A**) The mRNA and protein levels of LPR6 were detected by qRT-PCR and western blot assays respectively in LPR6 siRNAs or negative control siRNA-transfected cells. (**B**) The mRNA levels of osteogenic-related genes were detected by qRT-PCR assay on day 6 of osteogenic differentiation. (**C**) The protein levels of osteogenic-related genes were analyzed using western blot assays on day 6 of osteogenic differentiation. (**D, E**) ALP staining and ALP activity analyses were used to indicate the early differentiation on day 6 of osteogenic differentiation. (**F**) Alizarin red staining was performed to indicate calcium salt deposits on day 12. (**G, H**) qRT-PCR and Western blot assays analyzed the expression levels of CTNNB1. Scale bars: 200 μm. Quantitative data are presented as the mean ± S.D. (n =3). *P<0.05; **P<0.01; ***P<0.001.

